# DO TRAJECTORIES OF SELF-IDENTIFIED RETIREMENT STATUS PREDICT HEALTH AND COGNITIVE OUTCOMES?

**DOI:** 10.1093/geroni/igae098.2516

**Published:** 2024-12-31

**Authors:** Yun taek Oh, Jacqui Smith

**Affiliations:** University of Nevada Reno, Reno, Nevada, United States; University of Michigan, Ann Arbor, Michigan, United States

## Abstract

Few studies examined the ways older workers self-identify their retirement status (named ‘retirement identity’) over time before exiting the workforce. Examining the trajectories of retirement identity transitions – between “not retired”, “partly retired”, and “completely retired” – clarifies the heterogeneous outcomes found in ‘static’ retirement identity. In this study, we distinguish and categorize these trajectories using sequence analysis, investigate the common sociodemographic and economic characteristics for each trajectory using multinomial logistic regression, and examine the associations between these trajectories and the physical and mental health conditions of older workers using latent growth modeling. Drawing on a sample of respondents from the Health and Retirement Study (HRS) from 1992 to 2020, we use the first nine waves of the initial HRS birth cohort (born 1931-1936, recruited in 1992, N=1,487) and the Early Baby Boomers (born 1948-1953, recruited in 2004, N=1,017). We determine and name four distinct categories of trajectories – early, late, traditional, and partial transitions – based on the timing and sequence of transitions. The membership of these trajectories is strongly associated with the birth cohort, educational attainment, labor income from career jobs, and household wealth. The latent growth modeling reveals that workers in late or partial transition trajectories tend to have better initial physical, mental, and cognitive health and experience slower deterioration of these than those in early or traditional transition trajectories. These results suggest a close relationship between health and self-identification of retirement status that may influence the work attachment and timing of workforce exit.

